# Sex-based differences in clinical outcomes among patients with obesity and heart failure with preserved ejection fraction treated with tirzepatide

**DOI:** 10.1177/17539447261478928

**Published:** 2026-08-17

**Authors:** Ibrahim Mortada, Aaron W. Lee, Dalton Buckingham, Valerie Quach, James Rice, Shareef Mansour, Khaled Chatila, Mostafa Shalaby, Dietrich Jehle, Michael C. Boyars, Thomas A. Blackwell, Hani Jneid

**Affiliations:** 1 Department of Cardiovascular Medicine, 12338 University of Texas Medical Branch, Galveston, TX, USA; 2 John Sealy School of Medicine, 12338 University of Texas Medical Branch, Galveston, TX, USA; 3 Department of Internal Medicine, 12338 University of Texas Medical Branch, Galveston, TX, USA; 4 Department of Emergency Medicine, 12338 University of Texas Medical Branch, Galveston, TX, USA

**Keywords:** obesity, tirzepatide, heart failure, cardiovascular disease

## Abstract

**Background:**

Sex-related differences are well recognized in heart failure with preserved ejection fraction (HFpEF), but real-world data describing clinical outcomes by sex among patients with obesity and HFpEF treated with tirzepatide remain limited.

**Objectives:**

To compare observed hospitalization, cardiovascular, and gastrointestinal outcomes between male and female patients with obesity and HFpEF treated with tirzepatide.

**Design:**

Retrospective, propensity score-matched, within-treatment cohort study.

**Methods:**

A retrospective study was conducted using TriNetX database which included 110 healthcare organizations. Adults with obesity, HFpEF, and taking tirzepatide between June 1, 2022 and January 1, 2025 were identified. Males and females were balanced using 1:1 propensity score matching (n=1,654 per group). Primary outcome assessed included all-cause hospitalization. Secondary outcomes were all-cause mortality, HF exacerbation, major adverse cardiovascular events (MACE), incident atrial fibrillation, nausea/vomiting and diarrhea. Two prespecified falsification endpoints were used.

**Results:**

During 1-year follow-up, males had a lower hazard of all-cause hospitalization compared with females (HR 0.87, 95% CI 0.77–0.98). No significant differences were observed for all-cause mortality (HR 1.73, 95% CI 0.95–3.18), HF exacerbation (HR 1.14, 95% CI 0.90–1.44), MACE (HR 1.28, 95% CI 0.99–1.65), or incident atrial fibrillation (HR 1.21, 95% CI 0.61–2.43). Males experienced lower rates of nausea and vomiting (HR 0.50, 95% CI 0.37–0.67), while diarrhea was similar (HR 0.84, 95% CI 0.59–1.19). Falsification endpoints showed no significant associations.

**Conclusion:**

Among tirzepatide-treated patients with obesity and HFpEF, no statistically significant sex-associated differences were detected in major cardiovascular outcomes. Males had a modestly lower observed hazard of hospitalization and fewer coded nausea and vomiting events. Because this was a within-treatment observational comparison without an external comparator, these findings should not be interpreted as evidence of differential tirzepatide effectiveness by sex.

## Graphical abstract

**Graphical Abstract:**
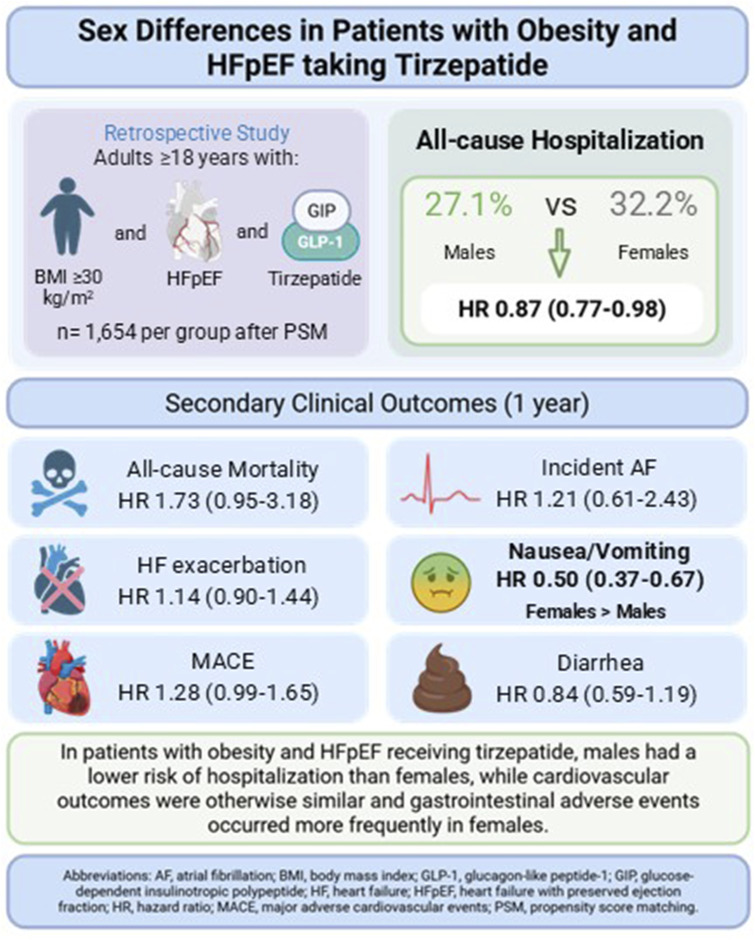

## Introduction

Heart failure with preserved ejection fraction (HFpEF) accounts for nearly half of all heart failure (HF) cases, and obesity, affecting up to 80% of individuals with HFpEF, remains one of the most prominent risk factors for its development, with particularly strong associations observed among women.^1,2^ Current guidelines suggest addressing obesity as a key comorbidity in HFpEF given its multifaceted associations with metabolic conditions such as diabetes mellitus, chronic kidney disease, and hypertension.^3^ Until recently, therapeutic options specifically targeting obesity-related HFpEF were limited. However, tirzepatide, a dual glucose-dependent insulinotropic polypeptide and glucose-like peptide-1 (GIP/GLP-1) receptor agonist has been shown to greatly reduce cardiovascular death and worsening HF in this patient population through inducing weight loss, reducing blood volume and blood pressure, decreasing systemic inflammation as well as cardio-renal end-organ damage.^4,5^ Despite growing evidence supporting tirzepatide’s effectiveness in obesity-related HFpEF, whether observed outcomes among men and women receiving tirzepatide differ remains incompletely described.^6^ Given the broad and overlapping burden of HFpEF and obesity, real-world assessments of tirzepatide may provide valuable insight into potential sex-specific differences in therapeutic response within this heterogeneous patient population. Despite growing use of tirzepatide in patients with obesity-related HFpEF, limited real-world data describe whether observed outcomes among treated patients differ by sex. Such differences may reflect variation in baseline HFpEF phenotype, symptom burden, healthcare utilization, medication tolerability, or treatment exposure rather than differential pharmacologic efficacy. Therefore, this study compared hospitalization, cardiovascular events, and gastrointestinal outcomes between male and female patients with obesity and HFpEF who were prescribed tirzepatide. Because no untreated or active comparator cohort was included, the analysis was designed as a within-treatment observational comparison rather than an evaluation of tirzepatide effectiveness or sex-based effect modification.

## Methods

This study was conducted as a retrospective analysis using data from the TriNetX Research Network,^7^ a global federated health research platform that provides access to de-identified electronic health records from participating healthcare organizations (HCOs). At the time of analysis, the TriNetX Research Network included approximately 110 contributing HCOs. Available data domains include patient demographics, diagnoses, procedures, medication exposures, laboratory values, and encounter information. All data are publicly available in the TriNetX Database through institutional agreement. The study was conducted in accordance with the Strengthening the Reporting of Observational Studies in Epidemiology (STROBE) guidelines^8^ and adhered to TriNetX publication standards. All clinical definitions, including diagnosis, medication, procedure, and laboratory codes used to define cohorts and outcomes, are provided in the Supplemental Material.

### Study cohort

Adult patients aged 18 years or older were eligible for inclusion. The study population consisted of patients with obesity and HFpEF who received tirzepatide between June 1, 2022, and January 1, 2025. Obesity was defined by a body mass index (BMI) ≥30 kg/m^2^ in conjunction with an ICD-10 diagnosis of overweight or obesity, and HFpEF was identified using ICD-10 diagnostic codes for diastolic HF. The validity of this approach is supported by Bates et al.,^9^ who demonstrated that ICD-10 coding for systolic and diastolic HF reliably distinguishes HFpEF and HF with reduced ejection fraction (EF) when an EF cutoff of 50% is applied. Patients were required to have documented initiation or administration of tirzepatide during the study period. Medication exposure in TriNetX reflects structured prescribing or administration records and does not confirm medication dispensing, adherence, dose, titration, persistence, discontinuation, or the reason for treatment cessation. Patients were stratified by sex into male and female cohorts using the TriNetX built-in Sex filter. For each cohort, the index date was defined as the first date on which all inclusion criteria were simultaneously met, including qualifying BMI, relevant diagnoses, and documentation of tirzepatide exposure. All inclusion criteria were required to be satisfied on or before the index date. Patients with missing key demographic information were excluded. No untreated or active comparator cohort was included because the objective was to compare observed outcomes between male and female patients receiving the same therapy. Accordingly, the study was not designed to estimate tirzepatide effectiveness, compare tirzepatide with alternative treatment strategies, or evaluate a sex-by-treatment interaction.

### Outcomes

Outcome assessment began 30 days after the index date and continued through 1 year after index. This prespecified 30-day landmark was used to separate baseline cohort identification and treatment initiation from subsequent outcome ascertainment and to reduce the likelihood that encounters occurring contemporaneously with tirzepatide initiation would be classified as follow-up outcomes. Patients were required to remain observable and free of the outcome under evaluation through day 30 to enter the corresponding time-to-event analysis. Patients were followed until the occurrence of an outcome event, censoring at the last recorded clinical encounter within the TriNetX network, or the end of the available follow-up window. The primary outcome evaluated in this analysis was all-cause hospitalization. Additional secondary outcomes included all-cause mortality, HF exacerbation, major adverse cardiovascular events (MACE), incident atrial fibrillation (AF), nausea/vomiting, diarrhea, traumatic brain injury (TBI) and carpal tunnel syndrome (CTS). All-cause hospitalization was defined by inpatient, short-stay, or emergency encounters. All-cause mortality was defined as death from any cause recorded in the electronic health records (EHR). HF exacerbation was defined using diagnostic codes for HF. Incident AF was defined using ICD-10 codes for unspecified, paroxysmal, or persistent AF and was evaluated as an incident outcome with exclusion of patients who had the diagnosis prior to the start of the time window. Gastrointestinal adverse events were defined by diagnostic codes for nausea and vomiting. TBI and CTS were included as falsification endpoints. For incident outcomes, patients with a documented history of the outcome before the follow-up window were excluded from the corresponding analysis.

### Propensity matching

To minimize confounding, propensity score matching (PSM) was performed between female and male cohorts. A 1:1 matching ratio was used, employing a greedy nearest-neighbor matching algorithm with a predefined caliper within the TriNetX platform. Prior to matching, patient records were randomized in accordance with TriNetX methodology to avoid order-dependent bias. Covariates included in the propensity score model were selected a priori based on clinical relevance and included demographic characteristics, baseline comorbidities, medication exposures, and available laboratory values. The propensity score model did not include direct measures of healthcare-seeking behavior, symptom perception, access to care, clinician admission thresholds, or institutional hospitalization practices because these variables were not reliably available in structured TriNetX data. Similarly, treatment adherence, tirzepatide dose, titration, persistence, and discontinuation could not be incorporated into the matching model. Covariate balance was assessed using standardized mean differences, with values <0.1 considered indicative of adequate balance. A complete list of covariates included in the matching process is provided in the Supplemental Table.

### Statistical analysis

All analyses were performed using the analytic framework available within the TriNetX platform. Descriptive statistics were generated for baseline characteristics before and after PSM. Risk estimates, including absolute risk differences, risk ratios, and odds ratios, were calculated where appropriate. Time-to-event outcomes were evaluated using Kaplan–Meier (KM) survival analyses, and hazard ratios with corresponding 95% confidence intervals were estimated using Cox proportional hazards models. Analyses were performed both before and after PSM, with post-matching results considered primary. Statistical significance was defined as a two-sided P value <0.05. All effect estimates represent associations between sex and observed outcomes within the tirzepatide-treated population. They should not be interpreted as estimates of the causal effect of tirzepatide or as evidence that tirzepatide treatment effects differ by sex. Hazard ratios were calculated comparing males with females, with females serving as the reference group. Because only aggregate TriNetX output was available, additional patient-level sensitivity analyses examining alternative landmark definitions could not be independently performed.

### IRB statement

This investigation was conducted using de-identified data obtained from the TriNetX Research Network and was deemed exempt from review by the University of Texas Medical Branch Institutional Review Board. The IRB determined that the study did not involve any direct interaction or intervention with human participants and that all data met the de-identification criteria outlined under §164.514(a) of the Health Insurance Portability and Accountability Act Privacy Rule.

## Results

### Cohort identification and matching

**Figure 1.**
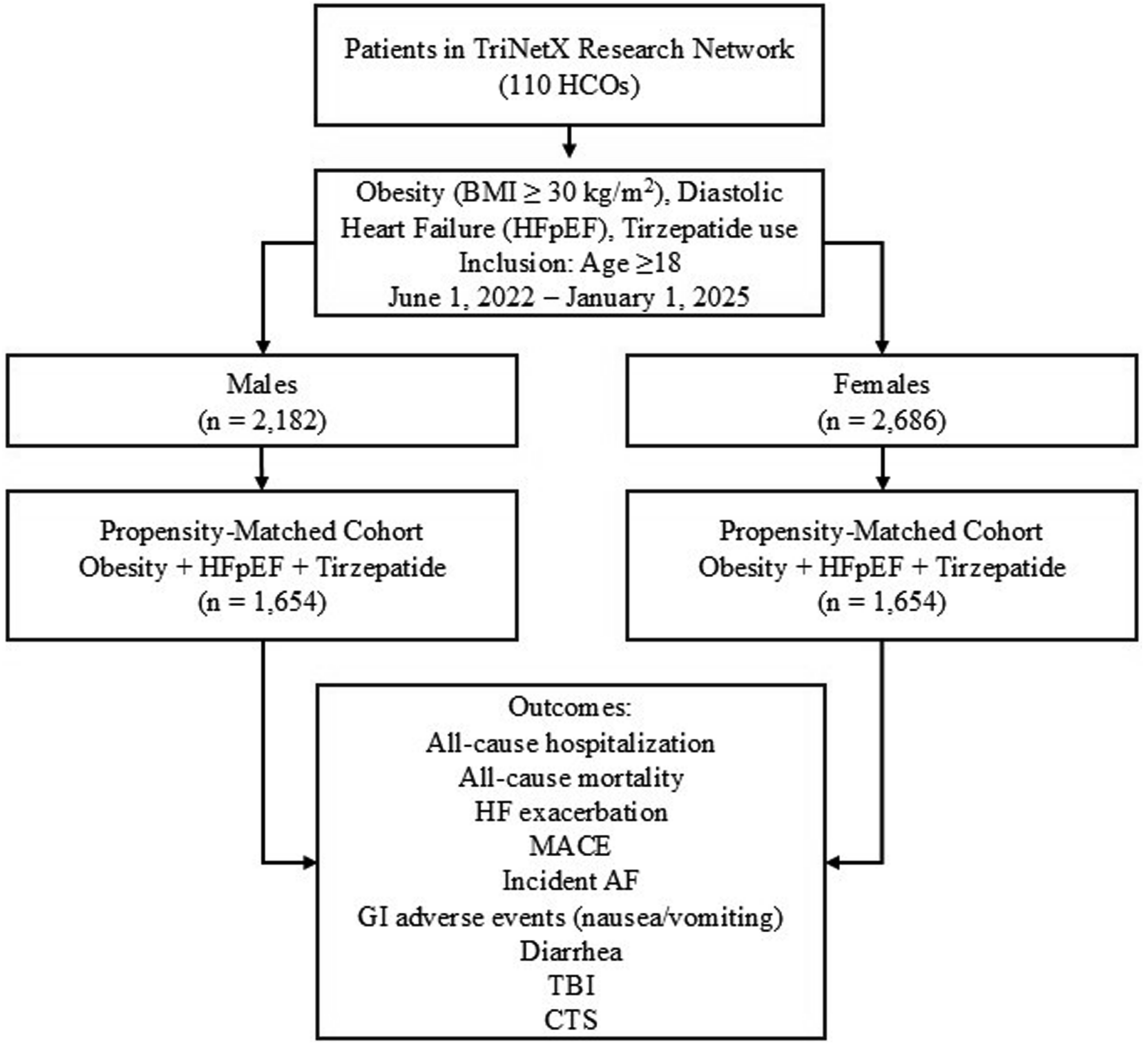
Cohort selection flowchart with propensity score–matched design comparing outcomes of Males vs Females with Obesity and HFpEF. Abbreviations: AF, atrial fibrillation; BMI, body mass index; CTS, carpal tunnel syndrome; GI, gastrointestinal; HF, heart failure; HFpEF, heart failure with preserved ejection fraction; HCO, healthcare organization; MACE, major adverse cardiovascular events; TBI, traumatic brain injury.

A total of 4,868 adult patients with obesity and HFpEF treated with tirzepatide met eligibility criteria, including 2,182 males and 2,686 females prior to PSM (Figure 1). Patients were stratified according to sex before matching. After 1:1 PSM, 1,654 females and 1,654 males remained in the final analytic cohorts (Figure 1). Post-matching assessment demonstrated improved balance across demographic characteristics, baseline comorbidities, medication exposures, and available laboratory parameters, with standardized mean differences generally below accepted thresholds, indicating effective overall covariate balance (Table 1).

**Table 1.** Baseline characteristics of sex-stratified tirzepatide-treated cohorts pre- and post-propensity score matching.

Characteristic	Pre-PSM				Post-PSM			
	Male	Female	SMD	P-value	Male	Female	SMD	P-value
Demographics								
Age at index, years (mean ± SD)	64.5 ± 11.2	65.2 ± 11.4	0.058	0.049	64.8 ± 11.3	64.9 ± 11.4	0.011	0.759
White, n (%)	1,682 (80.9)	1,813 (70.9)	0.236	<0.001	1,299 (78.5)	1,303 (78.8)	0.006	0.865
Hispanic or Latino, n (%)	80 (3.8)	105 (4.1)	0.013	0.657	65 (3.9)	69 (4.2)	0.012	0.724
Black or African American, n (%)	268 (12.9)	597 (23.3)	0.274	<0.001	254 (15.4)	256 (15.5)	0.003	0.923
Asian, n (%)	18 (0.9)	19 (0.7)	0.014	0.639	12 (0.7)	13 (0.8)	0.007	0.841
Diagnoses								
Chronic kidney disease (CKD), n (%)	694 (33.4)	827 (32.3)	0.022	0.447	517 (31.3)	544 (32.9)	0.035	0.315
Diabetes mellitus, n (%)	1,141 (54.9)	1,324 (51.8)	0.063	0.034	871 (52.7)	873 (52.8)	0.002	0.944
Ischemic heart diseases, n (%)	1,024 (49.3)	930 (36.4)	0.263	<0.001	717 (43.3)	714 (43.2)	0.004	0.916
Disorders of lipoprotein metabolism and other lipidemias, n (%)	1,514 (72.9)	1,801 (70.4)	0.054	0.069	1,184 (71.6)	1,183 (71.5)	0.001	0.969
Atrial fibrillation and flutter, n (%)	870 (41.9)	756 (29.6)	0.259	<0.001	607 (36.7)	598 (36.2)	0.011	0.745
Nicotine dependence, n (%)	247 (11.9)	282 (11.0)	0.027	0.361	192 (11.6)	185 (11.2)	0.013	0.702
Essential (primary) hypertension, n (%)	1,676 (80.7)	2,031 (79.4)	0.031	0.300	1,309 (79.1)	1,323 (80.0)	0.021	0.546
Old myocardial infarction, n (%)	242 (11.6)	199 (7.8)	0.131	<0.001	161 (9.7)	154 (9.3)	0.014	0.678
Cardiomyopathy, n (%)	391 (18.8)	316 (12.4)	0.179	<0.001	245 (14.8)	254 (15.4)	0.015	0.662
Cerebral infarction, n (%)	103 (5.0)	112 (4.4)	0.027	0.353	81 (4.9)	79 (4.8)	0.006	0.871
Medications								
Beta blockers/related, n (%)	1,230 (59.2)	1,379 (53.9)	0.106	<0.001	935 (56.5)	935 (56.5)	0.001	1
Calcium channel blockers, n (%)	716 (34.5)	846 (33.1)	0.029	0.326	566 (34.2)	549 (33.2)	0.022	0.532
ACE inhibitors, n (%)	365 (17.6)	340 (13.3)	0.118	<0.001	254 (15.4)	250 (15.1)	0.007	0.847
Angiotensin II inhibitor, n (%)	771 (37.1)	824 (32.2)	0.103	0.001	581 (35.1)	564 (34.1)	0.022	0.534
Insulins and analogues, n (%)	569 (27.4)	690 (27.0)	0.009	0.762	433 (26.2)	436 (26.4)	0.004	0.906
DPP-4 inhibitors, n (%)	52 (2.5)	73 (2.9)	0.022	0.461	37 (2.2)	38 (2.3)	0.004	0.907
SGLT2 inhibitors, n (%)	621 (29.9)	597 (23.3)	0.148	<0.001	448 (27.1)	436 (26.4)	0.016	0.637
Metformin, n (%)	362 (17.4)	375 (14.7)	0.075	0.011	256 (15.5)	262 (15.8)	0.01	0.774
Diuretics, n (%)	1,356 (65.3)	1,690 (66.1)	0.018	0.550	1,081 (65.4)	1,061 (64.1)	0.025	0.467
Potassium sparing/combinations diuretics, n (%)	557 (26.8)	626 (24.5)	0.053	0.071	419 (25.3)	420 (25.4)	0.001	0.968
HMG CoA reductase inhibitors, n (%)	1,182 (56.9)	1,358 (53.1)	0.076	0.010	894 (54.1)	899 (54.4)	0.006	0.861
Anticoagulants, n (%)	1,058 (50.9)	1,210 (47.3)	0.072	0.015	801 (48.4)	788 (47.6)	0.016	0.651
Laboratory Values								
HbA1c (mean ± SD)	6.9 ± 1.7	6.7 ± 1.6	0.084	0.020	6.8 ± 1.6	6.8 ± 1.7	0.019	0.657
HbA1c 3–6%, n (%)	597 (28.7)	818 (32.0)	0.071	0.017	502 (30.4)	491 (29.7)	0.015	0.676
HbA1c 6–9%, n (%)	908 (43.7)	987 (38.6)	0.104	<0.001	691 (41.8)	666 (40.3)	0.031	0.377
HbA1c 9–12%, n (%)	192 (9.2)	207 (8.1)	0.041	0.167	135 (8.2)	140 (8.5)	0.011	0.753
HbA1c ≥12%, n (%)	41 (2.0)	46 (1.8)	0.013	0.664	29 (1.8)	34 (2.1)	0.022	0.525
BMI, kg/m^2^ (mean ± SD)	40.3 ± 7.6	41.8 ± 8.5	0.179	<0.001	40.7 ± 7.7	41.1 ± 8.5	0.044	0.220
BMI 30–35 kg/m^2^, n (%)	632 (30.4)	676 (26.4)	0.088	0.003	489 (29.6)	477 (28.8)	0.016	0.646
BMI 35–40 kg/m^2^, n (%)	873 (42.0)	945 (37.0)	0.104	<0.001	657 (39.7)	660 (39.9)	0.004	0.915
BMI ≥40 kg/m^2^, n (%)	1,064 (51.2)	1,505 (58.9)	0.154	<0.001	894 (54.1)	888 (53.7)	0.007	0.834
LVEF, % (mean ± SD)	56.6 ± 11.2	59.8 ± 10.3	0.295	<0.001	58.3 ± 9.3	59.2 ± 10.6	0.099	0.307
LVEF ≤50%, n (%)	58 (2.8)	38 (1.5)	0.09	0.002	31 (1.9)	29 (1.8)	0.009	0.794
LVEF >50%, n (%)	238 (11.5)	274 (10.7)	0.023	0.426	192 (11.6)	189 (11.4)	0.006	0.870
Hemoglobin, g/dL (mean ± SD)	13.7 ± 2.0	12.5 ± 1.7	0.666	<0.001	13.6 ± 2.1	12.7 ± 1.6	0.48	<0.001
Hemoglobin ≤11 g/dL, n (%)	338 (16.3)	688 (26.9)	0.261	<0.001	308 (18.6)	301 (18.2)	0.011	0.753
Hemoglobin >11 g/dL, n (%)	1,560 (75.1)	1,932 (75.6)	0.011	0.703	1,236 (74.7)	1,228 (74.2)	0.011	0.750
BNP, pg/mL (mean ± SD)	393.4 ± 1192.9	386.2 ± 1482.5	0.005	0.938	408.7 ± 1331.1	481.6 ± 1848.2	0.045	0.577
BNP 0–150 pg/mL, n (%)	253 (12.2)	329 (12.9)	0.021	0.480	200 (12.1)	202 (12.2)	0.004	0.915
BNP 150–300 pg/mL, n (%)	98 (4.7)	108 (4.2)	0.024	0.419	78 (4.7)	79 (4.8)	0.003	0.935
BNP 300–450 pg/mL, n (%)	44 (2.1)	77 (3.0)	0.057	0.058	37 (2.2)	29 (1.8)	0.035	0.320
BNP 450–600 pg/mL, n (%)	34 (1.6)	37 (1.4)	0.015	0.602	23 (1.4)	19 (1.1)	0.022	0.534
BNP ≥600 pg/mL, n (%)	71 (3.4)	67 (2.6)	0.047	0.113	49 (3.0)	50 (3.0)	0.004	0.919
NT-proBNP, pg/mL (mean ± SD)	1499.3 ± 3828.9	1341.5 ± 3366.8	0.044	0.543	1404.7 ± 3608.0	1374.0 ± 3482.1	0.009	0.921
NT-proBNP 0–300 pg/mL, n (%)	147 (7.1)	230 (9.0)	0.071	0.017	123 (7.4)	124 (7.5)	0.002	0.947
NT-proBNP 300–600 pg/mL, n (%)	89 (4.3)	130 (5.1)	0.038	0.201	79 (4.8)	70 (4.2)	0.026	0.451
NT-proBNP 600–900 pg/mL, n (%)	55 (2.6)	76 (3.0)	0.02	0.506	45 (2.7)	45 (2.7)	0.001	1
NT-proBNP 900–1200 pg/mL, n (%)	37 (1.8)	52 (2.0)	0.019	0.532	31 (1.9)	32 (1.9)	0.004	0.899
NT-proBNP ≥1200 pg/mL, n (%)	114 (5.5)	140 (5.5)	<0.001	0.987	91 (5.5)	89 (5.4)	0.005	0.878

Abbreviations: ACE, angiotensin-converting enzyme; ARB, angiotensin II receptor blocker; BNP, B-type natriuretic peptide; BMI, body mass index; CKD, chronic kidney disease; DPP-4, dipeptidyl peptidase-4; HbA1c, hemoglobin A1c; HMG-CoA, 3-hydroxy-3-methylglutaryl–coenzyme A; LVEF, left ventricular ejection fraction; NT-proBNP, N-terminal pro–B-type natriuretic peptide; PSM, propensity score matching; SD, standard deviation; SGLT2, sodium–glucose cotransporter 2; SMD, standardized mean difference.

### Outcomes

**Figure 2.**
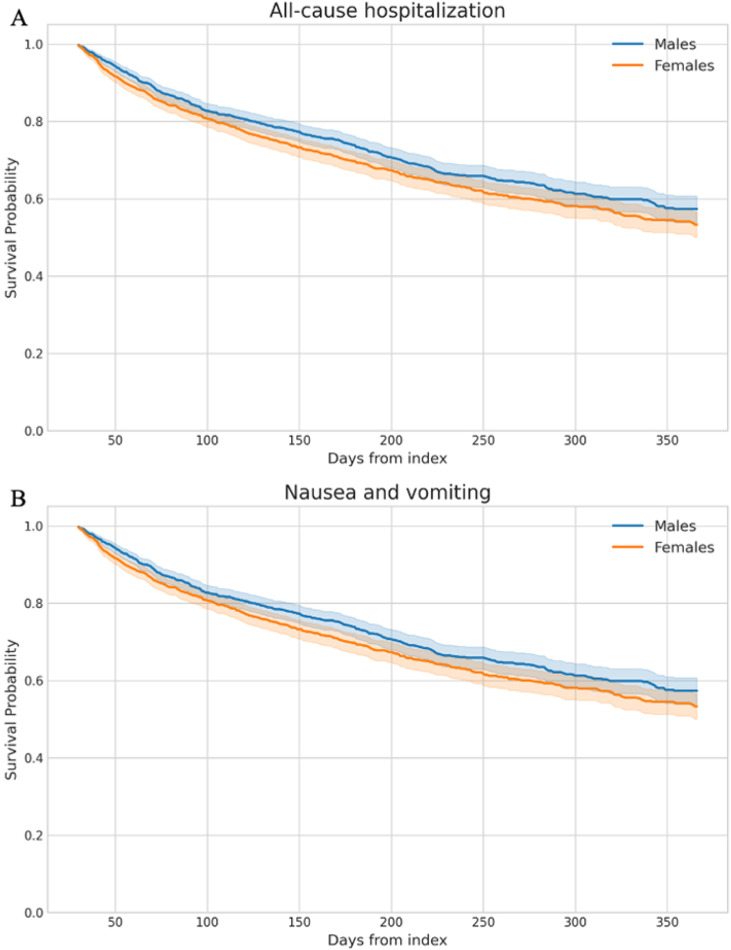
Kaplan-Meier curves showing survival analysis for all-cause hospitalization (A), and Nausea and vomiting (B).

The primary outcome of all-cause hospitalization occurred less frequently among males compared with females in the matched cohorts (27.1% vs, 32.2% respectively) (Table 2). Survival analysis demonstrated a significantly lower hazard of hospitalization among males relative to females, with KM curves demonstrating progressive divergence over follow-up and males having a higher probability of remaining hospitalization-free during follow-up (HR 0.87, 95% CI 0.76–0.98) (Figure 2(A)).

**Table 2.** Clinical outcomes among male and female patients treated with tirzepatide after PSM.

Outcome	Males (%) N=1,654	Females (%) N=1,654	ARD (95% CI)	RR (95% CI)	OR (95% CI)	HR (95% CI)	Log-rank P-value
All-cause hospitalization	448 (27.1)	533 (32.2)	−0.051 (−0.082 to −0.020)	0.84 (0.76–0.93)	0.78 (0.67–0.91)	0.87 (0.77–0.98)	0.026
All-cause mortality	27 (1.6)	17 (1.0)	0.006 (−0.002 to 0.014)	1.59 (0.87–2.90)	1.60 (0.87–2.94)	1.73 (0.95–3.18)	0.072
HF exacerbation	147 (8.9)	138 (8.3)	0.005 (−0.014 to 0.025)	1.07 (0.85–1.33)	1.07 (0.84–1.37)	1.14 (0.90–1.44)	0.272
MACE	126 (7.6)	106 (6.4)	0.012 (−0.005 to 0.029)	1.19 (0.93–1.53)	1.20 (0.92–1.57)	1.28 (0.99–1.65)	0.062
Incident AF	16 (1.7)	16 (1.6)	0.001 (−0.011 to 0.013)	1.06 (0.53–2.10)	1.06 (0.53–2.13)	1.21 (0.61–2.43)	0.582
Nausea/Vomiting	63 (3.8)	131 (7.9)	−0.041 (−0.057 to −0.025)	0.48 (0.36–0.64)	0.46 (0.34–0.63)	0.50 (0.37–0.67)	<0.001
Diarrhea	56 (3.4)	71 (4.3)	−0.009 (−0.022 to 0.004)	0.79 (0.56–1.11)	0.78 (0.55–1.12)	0.84 (0.59–1.19)	0.323
TBI	11 (0.7)	19 (1.1)	−0.005 (−0.011 to 0.002)	0.58 (0.28–1.21)	0.58 (0.27–1.21)	0.61 (0.29–1.29)	0.194
CTS	25 (1.5)	34 (2.1)	—	—	—	0.77 (0.46–1.30)	0.330

Abbreviations: AF, atrial fibrillation; ARD, absolute risk difference; CI, confidence interval; CTS, carpal tunnel syndrome; GI, gastrointestinal; HF, heart failure; HR, hazard ratio; MACE, major adverse cardiovascular events; OR, odds ratio; PSM, propensity score matching; RR, risk ratio; TBI, traumatic brain injury.

Among secondary cardiovascular outcomes, all-cause mortality occurred infrequently and was numerically higher among males compared with females (1.6% vs 1.0%), although this difference did not reach statistical significance (HR 1.72, 95% CI 0.94–3.23). HF exacerbation occurred at similar rates among males compared with females treated with tirzepatide (8.9% vs 8.3%, respectively), and no significant difference in the hazard of HF exacerbation was observed (HR 1.14, 95% CI 0.90–1.43). Similarly, MACE occurred at comparable rates among males compared with females (7.6% vs 6.4%), with no statistically significant difference in the hazard of MACE among males relative to females (HR 1.28, 95% CI 0.99–1.67). Incident AF occurred infrequently after exclusion of patients with prior AF and was observed at comparable rates among males compared with females (1.7% vs 1.6%, respectively), with no significant difference in hazard among males relative to females (HR 1.22, 95% CI 0.61–2.44).

Gastrointestinal outcomes demonstrated some sex-based differences. Nausea and vomiting events occurred less frequently among males compared with females (3.8% vs 7.9%), corresponding to a significantly lower hazard among males relative to females (HR 0.50, 95% CI 0.37–0.68). Kaplan–Meier analysis demonstrated early separation of the curves, with males exhibiting a lower probability of experiencing nausea and vomiting events compared with females (Figure 2(B)). Diarrhea occurred in 3.4% of males compared with 4.3% of females, although no statistically significant difference in hazard was observed (HR 0.84, 95% CI 0.59–1.19).

Consistent with expectations for falsification endpoints, TBI events were rare and occurred at similar rates among males compared with females (0.7% vs 1.1%), with no statistically significant difference in hazard (HR 0.61, 95% CI 0.29–1.30). Similarly, CTS occurred at comparable rates among males and females, with no statistically significant difference between groups (HR 0.78, 95% CI 0.46–1.30), although these findings provide only limited reassurance regarding broad systematic differences between the matched cohorts.

## Discussion

This retrospective within-treatment analysis compared observed outcomes between male and female patients with obesity and HFpEF who were treated with tirzepatide. No statistically significant sex-associated differences were detected in all-cause mortality, HF exacerbation, MACE, or incident atrial fibrillation. Males had a modestly lower observed hazard of all-cause hospitalization and fewer coded nausea and vomiting events than females. These findings characterize outcome patterns within a tirzepatide-treated population but do not establish that tirzepatide is more or less effective in either sex. Without an untreated or active comparator group, the observed associations cannot be separated from broader sex-related differences in HFpEF phenotype, symptom burden, healthcare utilization, treatment exposure, or clinical documentation.

All-cause hospitalization represents a clinically meaningful endpoint in HF populations, as hospitalization events are associated with accelerated functional decline, increased mortality risk, and substantial healthcare utilization.^10^ The higher hospitalization rate observed among females in this cohort may reflect differences not fully captured by propensity matching. HFpEF is more prevalent among women, who account for the majority of HFpEF cases and hospitalizations.^10^ Females with HFpEF frequently demonstrate greater symptom burden, impaired exercise capacity, and reduced quality of life compared with men despite similar objective measures of cardiac dysfunction.^11^ Emerging data suggest that HFpEF represents a sex-specific syndrome in which women exhibit greater ventricular and vascular stiffness, increased microvascular dysfunction, and heightened systemic inflammation relative to men.^12,13^ These biological differences could plausibly contribute to symptom burden and healthcare utilization; however, this interpretation remains speculative because detailed physiologic measures, symptom assessments, access-to-care variables, and hospitalization decision factors were unavailable.

The absence of statistically significant sex-associated differences in cardiovascular outcomes within this treated cohort is broadly consistent with subgroup findings from randomized trials of incretin-based therapies, although the present study cannot directly assess treatment efficacy or compare treatment effects by sex. In the landmark tirzepatide trial evaluating patients with obesity-related HFpEF, tirzepatide significantly reduced the composite of cardiovascular death or worsening HF while improving weight and cardiometabolic parameters.^14^ Although sex-specific outcomes were not a primary focus of that study, the overall cardiometabolic benefits observed were consistent across diverse patient subgroups. Similarly, semaglutide has been shown to improve HF symptoms, functional status, and weight loss among patients with obesity-related HFpEF.^15^ Subsequent analyses of the STEP-HFpEF trials demonstrated that improvements in Kansas City Cardiomyopathy Questionnaire scores were comparable between women and men despite greater weight loss among women.^16^ These trial findings suggest that cardiometabolic therapies may have broadly consistent effects across sexes; however, the present within-treatment analysis was not designed to estimate sex-specific treatment benefit.

Tirzepatide has been proposed to influence obesity-related HFpEF through weight reduction, improved insulin sensitivity, reductions in systemic inflammation, and favorable effects on endothelial function and cardiac loading conditions.^12^ However, the present analysis lacked serial weight change, detailed physiologic measurements, and longitudinal treatment-exposure data and therefore could not evaluate these mechanisms or determine whether they differed by sex.

The higher incidence of documented nausea and vomiting among females in this cohort is consistent with prior reports of sex-related differences in gastrointestinal symptom reporting during incretin-based therapy. Gastrointestinal adverse events remain the most common side effects associated with GLP-1 receptor agonists and related therapies, with nausea reported in approximately 25–44% of patients.^17^ Previous analyses have demonstrated that women are more likely than men to report gastrointestinal symptoms during GLP-1 receptor agonist therapy.^18^ In a head-to-head trial comparing tirzepatide with semaglutide, gastrointestinal events leading to treatment discontinuation were less frequent with tirzepatide (2.7% versus 5.6%).^19^ The higher rate of documented nausea and vomiting among females may reflect differences in symptom burden, clinical reporting, dose exposure, or treatment persistence. Because these factors were unavailable or incompletely captured, the finding should not be interpreted as confirmed sex-related differences in medication tolerability. These observations support further study of sex-related differences in gastrointestinal symptom burden and clinical documentation during treatment.

Real-world analyses such as this study complement randomized trials by evaluating observed outcomes in broader patient populations and routine clinical practice settings where comorbidity burden, medication adherence, and healthcare utilization patterns may differ from controlled trial environments. The absence of significant associations for falsification endpoints provides limited reassurance against broad systematic differences between the matched cohorts. However, because TBI and CTS do not closely share the confounding structure of hospitalization or cardiovascular outcomes, these findings do not exclude outcome-specific residual confounding.

From a clinical perspective, the data did not identify a statistically significant difference in major cardiovascular event rates between male and female patients receiving tirzepatide. The modest hospitalization and nausea/vomiting associations may warrant further study, but they should not be interpreted as evidence of sex-specific tirzepatide effectiveness because differences in symptom burden, healthcare utilization, dose, adherence, and treatment persistence were not fully captured.

## Limitations

Several limitations should be considered when interpreting these findings. The study included only patients treated with tirzepatide and did not include an untreated or active comparator cohort. Therefore, it cannot estimate the effectiveness of tirzepatide, determine whether tirzepatide altered outcomes relative to another treatment strategy, or establish whether treatment effects differed by sex. Observed differences may instead reflect underlying sex-related differences in HFpEF, healthcare utilization, symptom reporting, treatment exposure, or other unmeasured characteristics. This study was conducted using a retrospective observational design based on EHR data from the TriNetX research network. Although PSM was used to balance measured covariates between male and female cohorts, the possibility of residual confounding from unmeasured variables cannot be excluded, as is inherent to observational research.^20^ In particular, the available data did not capture healthcare-seeking behavior, symptom perception, access to care, clinician decision-making, admission thresholds, or institutional hospitalization practices. These factors may differ by sex and may be especially relevant to interpretation of the hospitalization outcome. Additionally, clinical data within the TriNetX platform are derived from structured EHR documentation and rely on diagnostic and procedural coding. While ICD-based definitions of cardiovascular conditions have been validated in prior work, variation in coding practices across participating HCOs may introduce some degree of misclassification.^21,22^

Outcome and covariate definitions in this study were based on structured diagnostic and procedural coding. Although HF can be identified using administrative data, prior studies have shown that the validity of coded HF diagnoses varies across data sources and coding strategies, introducing the possibility of misclassification.^23,24^ In addition, several granular physiologic variables relevant to HFpEF—including detailed diastolic function measures, filling pressures, and exercise capacity—are not consistently available in EHR-based datasets, limiting adjustment for disease severity. Accordingly, proposed biological explanations for the observed sex-associated differences remain speculative because the dataset lacked detailed physiologic measures, serial weight change, and longitudinal treatment-exposure information. Medication exposure in TriNetX reflects documented prescribing or administration rather than confirmed medication dispensing or use. Accordingly, the data do not reliably establish serial weight change, adherence, dose, titration pattern, treatment persistence, discontinuation, or reasons for stopping therapy. These limitations are relevant when interpreting both clinical outcomes and coded gastrointestinal events, because actual treatment exposure may have differed between male and female patients.

Follow-up began at a prespecified 30-day landmark after the index date. Because sex was fixed at baseline and all patients initiated tirzepatide before follow-up, classic exposure-related immortal time bias was not expected. However, the landmark excluded events occurring during the first 30 days and conditioned inclusion on remaining observable and event-free through the start of follow-up, which may introduce selection bias and limits inference regarding early treatment-period outcomes. Additional patient-level sensitivity analyses using alternative landmark definitions could not be independently performed because only aggregate TriNetX output was available.

The primary outcome of hospitalization may also be influenced by sex-related differences in symptom burden and healthcare utilization. Females with HFpEF often have greater symptom limitation and worse exercise capacity than males, even when resting cardiac measures appear similar.^11,13^ Finally, because this analysis was observational, the findings should be interpreted as associations rather than causal effects. Future studies incorporating untreated or active comparator groups and formal sex-by-treatment interaction analyses are needed to determine whether the effects of tirzepatide truly differ by sex.

## Conclusion

In this within-treatment observational analysis of patients with obesity and HFpEF treated with tirzepatide, no statistically significant sex-associated differences were detected in mortality, HF exacerbation, MACE, or incident atrial fibrillation. Males had a modestly lower observed hazard of all-cause hospitalization and fewer coded nausea and vomiting events than females. Because no untreated or active comparator group was included, these findings do not establish differential tirzepatide effectiveness by sex and may reflect residual differences in HFpEF phenotype, healthcare utilization, symptom reporting, or treatment exposure. Comparative studies with formal interaction testing are needed to determine whether tirzepatide-associated treatment effects differ between men and women.

## Supplemental material

Supplemental Material - Distinguishing Pedohebephebophilic Actors and Non-Actors: A Meta-Analysis Supplemental Material for Sex-based differences in clinical outcomes among patients with obesity and heart failure with preserved ejection fraction treated with tirzepatide by Ibrahim Mortada, Aaron W. Lee, Dalton Buckingham, Valerie Quach, James Rice, Shareef, Mansour, Khaled Chatila, Mostafa Shalaby, Dietrich Jehle, Michael C. Boyars, Thomas A. Blackwell, Hani Jneid in Therapeutic Advances in Cardiovascular Disease

## Data Availability

All data are publicly available in the TriNetX Database through institutional agreement.

## ORCID iDs

Ibrahim Mortada https://orcid.org/0009-0006-9215-880X

Aaron W. Lee https://orcid.org/0009-0009-0979-4904

Dalton Buckingham https://orcid.org/0009-0005-7805-4353

Valerie Quach https://orcid.org/0009-0002-3430-1672

Shareef Mansour https://orcid.org/0000-0001-7929-8839

Khaled Chatila https://orcid.org/0000-0003-3262-3096

Mostafa Shalaby https://orcid.org/0009-0008-7258-6887

Dietrich Jehle https://orcid.org/0009-0002-6006-3903

Michael C. Boyars https://orcid.org/0000-0003-2606-4283

Thomas A. Blackwell https://orcid.org/0009-0009-0659-2100

Hani Jneid https://orcid.org/0000-0002-8754-358X
